# *Bothrops moojeni* L-amino acid oxidase induces apoptosis and epigenetic modulation on Bcr-Abl^+^ cells

**DOI:** 10.1590/1678-9199-JVATITD-2020-0123

**Published:** 2020-12-14

**Authors:** Sandra Mara Burin, Maira da Costa Cacemiro, Juçara Gastaldi Cominal, Rone Aparecido De Grandis, Ana Rita Thomazela Machado, Flavia Sacilotto Donaires, Adelia Cristina Oliveira Cintra, Luciana Ambrosio, Lusânia Maria Greggi Antunes, Suely Vilela Sampaio, Fabíola Attié de Castro

**Affiliations:** 1 Department of Clinical Analyses, Toxicology and Food Sciences, School of Pharmaceutical Sciences, University of São Paulo (USP), Ribeirão Preto, SP, Brazil.; 2 Department of Internal Medicine, Ribeirão Preto Medical School, University of São Paulo (USP), Ribeirão Preto, SP, Brazil.

**Keywords:** Apoptosis, MicroRNA, Chronic myeloid leukemia, Snake toxins, BmooLAAO-I, Bothrops moojeni

## Abstract

**Background:**

Resistance to apoptosis in chronic myeloid leukemia (CML) is associated with constitutive tyrosine kinase activity of the Bcr-Abl oncoprotein. The deregulated expression of apoptosis-related genes and alteration in epigenetic machinery may also contribute to apoptosis resistance in CML. Tyrosine kinase inhibitors target the Bcr-Abl oncoprotein and are used in CML treatment. The resistance of CML patients to tyrosine kinase inhibitors has guided the search for new compounds that may induce apoptosis in Bcr-Abl^+^ leukemic cells and improve the disease treatment.

**Methods:**

In the present study, we investigated whether the L-amino acid oxidase isolated from *Bothrops moojeni* snake venom (BmooLAAO-I) (i) was cytotoxic to Bcr-Abl^+^ cell lines (HL-60.Bcr-Abl, K562-S, and K562-R), HL-60 (acute promyelocytic leukemia) cells, the non-tumor cell line HEK-293, and peripheral blood mononuclear cells (PBMC); and (ii) affected epigenetic mechanisms, including DNA methylation and microRNAs expression *in vitro*.

**Results:**

BmooLAAO-I induced ROS production, apoptosis, and differential DNA methylation pattern of regulatory apoptosis genes. The toxin upregulated expression of the pro-apoptotic genes *BID* and *FADD* and downregulated *DFFA* expression in leukemic cell lines, as well as increased miR-16 expression - whose major predicted target is the anti-apoptotic gene *BCL2* - in Bcr-Abl^+^ cells.

**Conclusion:**

BmooLAAO-I exerts selective antitumor action mediated by H_2_O_2_ release and induces apoptosis, and alterations in epigenetic mechanisms. These results support future investigations on the effect of BmooLAAO-I on *in vivo* models to determine its potential in CML therapy.

## Background

Chronic myeloid leukemia (CML) is a myeloproliferative neoplasm characterized by the Bcr-Abl oncoprotein expression, which promotes the uncontrolled cell proliferation and resistance to apoptosis [1,2]. The CML patients are currently treated with tyrosine kinase inhibitors (TKI) that target the Bcr-Abl oncoprotein, including imatinib mesylate, dasatinib, and nilotinib. Despite the high rates of molecular response to TKI therapy, many patients may acquire resistance to this therapy [3,4] due to mutations in the kinase domain of the Bcr-Abl oncoprotein, and to Bcr-Abl-independent mechanisms that mediate activation of alternative cell survival signaling pathways, which are associated with epigenetic and apoptotic deregulation [5].

Epigenetic deregulation plays a significant role on the development, maintenance and progression of different neoplasms, including the hematological neoplasms [6-8]. Epigenetic deregulation involves gene expression changes that promote heritable phenotype alterations with preserved DNA sequence [9]. DNA methylation and microRNA (miRNA) expression are key epigenetic regulation mechanisms for silencing gene expression [10].

DNA methylation regulates gene transcription and maintain genome stability [11]. The presence of gene-specific hypermethylation in the promoter region results in transcriptional repression of genes in different types of neoplastic cells [2,6,12].

miRNA is an endogenous small non-coding RNA molecule capable of inhibiting the gene expression [13] mostly by miRNA-mRNA binding, which could result in cleavage or degradation of the expressed mRNA [14,15]. miRNA can control a variety of biological processes, such as cell differentiation, proliferation, and apoptosis, but abnormal miRNA expression is associated with pathogenesis of solid tumors and hematological neoplasms such as lymphoma, acute and chronic lymphocytic leukemia, acute promyelocytic leukemia, and CML [16,17].

The miRNA molecules that mediate regulation of apoptosis-related genes expression, known as apoptomiR, are deregulated in CML. Our research team has reported the differential apoptomiR expression in CML patients in chronic and accelerated phase, as well as in patients resistant to the TKI imatinib mesylate [17]. Patients resistant to TKI therapy present an apoptomiR expression profile linked to overexpression of anti-apoptotic genes and dowregulation of pro-apoptotic genes [17]. The apoptomiR miR-15a and miR-16, which are involved in the control of the Bcl-2 protein expression, are also low expressed in Bcr-Abl^+^ leukemic cells [18,19]. Thus, it is relevant to seek new compounds that induce apoptosis and modulate the CML epigenetic machinery to increase sensitivity of leukemic cells to TKI therapy.

In this context, L-amino acid oxidases (LAAO) isolated from snake venom (SV-LAAO) have attracted great interest of the scientific and medical fields due to their antitumor potential [20-23]. The mechanisms by which SV-LAAO exerts their biological actions such as cytotoxicity and apoptosis induction remain unclear, but there are evidences that they are mediated by H_2_O_2_ production. H_2_O_2_ is a reactive oxygen species (ROS) that destabilizes mitochondrial membrane and induces cell death [22,24-27]. 

We have recently reported the antitumor potential of LAAO from *Bothrops pirajai* (BpirLAAO-I) and *Calloselasma rhodostoma* (CR-LAAO) venom, which induce apoptosis in Bcr-Abl^+^ cell lines [28-30]. The LAAO isolated from *Bothrops moojeni* (BmooLAAO-I) exerts antitumor action on Ehrlich ascites carcinoma cells and HL-60 acute promyelocytic leukemia cells [31]. The long-term enzymatic stability of BmooLAAO-I makes it possible to assess its pharmacological effects [32]. The present study examined whether BmooLAAO-I affected the apoptotic and epigenetic machineries of Bcr-Abl^+^ cell lines resistant and responsive to imatinib mesylate.

## Methods

### BmooLAAO-I isolation

BmooLAAO-I was isolated from a *B. moojeni* snake venom sample that was kindly donated by the Center for the Study of Venoms and Venomous Animals (CEVAP) of São Paulo State University (UNESP - Botucatu, SP, Brazil), and stored at − 20 °C. 

Crude venom (200 mg) was purified according to the protocol reported by Stábeli and collegues [31], with minor modifications. Initially, unpurified venom sample was concentrated by ultrafiltration using an AMICON^®^ apparatus equipped with a 10,000-Da cutoff membrane. The concentrated fraction was purified by hydrophobic chromatography on CM-Sepharose and Phenyl-Sepharose CL-4B columns (1.0×26 cm) previously equilibrated with 0.02 M Tris-HCl buffer, pH 7.4. Elution was carried out using a reverse linear NaCl gradient (4-0 M) at a flow rate of 72 mL/h, at 25 ºC, and fractions of 3.0 mL were collected. The fractions with LAAO activity were pooled, concentrated by ultrafiltration using a 30,000-Da cutoff membrane, and submitted to a third purification step by affinity chromatography on a Benzamidine Sepharose column (1.8×10 cm) previously equilibrated with 20 mM Tris-HCl, pH 7.4. The sample was eluted using a step gradient of 20 mM Tris-HCl supplemented with 1.0 M NaCl, pH 7.4, at a flow rate of 1 mL/h. Fractions of 3 mL were collected and followed by recording absorbance at 280 nm. The LAAO-active fraction was collected, concentrated by ultrafiltration using a 30,000-Da cutoff membrane, and stored at 4 °C for subsequent analysis. The purification parameters of BmooLAAO-I were summarized in the Additional file 1, it yielded 2.0% protein, specific activity of 2,806 U/mg with 12.3-purification fold.

The purity of the isolated BmooLAAO-I sample was analyzed by 12% (w/v) SDS-PAGE and high-performance liquid chromatography using a C18 reverse phase column (0.46×25 cm) equilibrated with 0.1% (v/v) trifluoroacetic acid. Elution was performed at a flow rate of 1 mL/min, for 90 min, using a concentration gradient (0-100%, v/v) of 70% acetonitrile in 0.1% trifluoroacetic acid (v/v). The fold purity of BmoLAAO-I was 12.3-fold. 

### Determination of enzymatic activity of BmooLAAO-I

Total protein concentration was determined using the BCA Protein Assay Kit^®^ (Thermo Fischer Scientific, Rockford, IL, USA), according to the manufacturer’s instructions. The BmooLAAO-I specific enzymatic activity was determined, prior to assays, by spectrophotometry using L-leucine as substrate [33].

### Cell lines

We used the cell lines HEK-293 (embryonic epithelial cells of human kidney), HL-60 (human promyelocytic leukemia cells), HL-60.Bcr-Abl (HL-60 infected with retrovirus carrying the *BCR-ABL1* gene), K562-S (imatinib mesylate-sensitive Bcr-Abl^+^ cells), and K562-R (imatinib mesylate-resistant Bcr-Abl^+^ cells). The K562-R and K562-S cells were obtained from a human CML patient in blastic phase. The tumor cell lines were kindly provided by Dr. Gustavo P. Amarante-Mendes (Institute of Biomedical Sciences, University of São Paulo, São Paulo, SP, Brazil), while HEK-293 cells were kindly provided by Dr. Andreia Machado Leopoldino (School of Pharmaceutical Sciences of Ribeirão Preto, University of São Paulo, Ribeirão Preto, SP, Brazil). All the leukemic cell lines were cultured in complete RPMI (Roswell Park Memorial Institute) 1640 medium, while HEK-293 cells were cultured in complete DMEM (Dulbecco’s Modified Eagle Medium); both media were supplemented with 10% fetal bovine serum and 1% penicillin/streptomycin, and all the cell lines were cultured at 37 °C, under 5% CO_2_.

### Isolation of peripheral blood mononuclear cells

Peripheral blood samples from three human healthy donors aged between 20 and 35 years old were collected into vaccum tubes containing EDTA (BD Vacutainer^®^). Peripheral blood mononuclear cells (PBMC) were isolated using the Ficoll-Hypaque density gradient method (Ficoll^®^ Paque Plus, GE Healthcare), according to the manufacturer’s instructions. Cell viability was determined using the trypan blue exclusion assay. 

The Human Research Ethics Committee of the School of Pharmaceutical Sciences of Ribeirão Preto, University of São Paulo, Brazil, approved the study protocol (CAAE number 55672816.6.000.5403). All healthy volunteers signed the informed consent form and agreed to participate in the study.

### Cytotoxicity assay

Cell viability was assessed using the 3-(4,5-dimethylthiazol-2-yl)-2,5-diphenyltetrazolium bromide (MTT) method described by Mosmann [34]. The five cell lines and PBMC cells (2×10^4^) were cultured in 180 µL of RPMI 1640 complete medium in 96-well plates and treated with BmooLAAO-I (0.000765-0.392 μg/mL) for 24 h. The cell lines were cultured with BmooLAAO-I in the presence or not of catalase (100-400 μg/mL) (Sigma-Aldrich, St. Louis, MO, USA) [22,35,36]. Untreated cells were used as the negative control. Next, 20 μL of MTT solution (5 mg/mL) were added to each well, and the plates were incubated (4 h, 37 °C) and centrifuged (660 ×*g*, 3 min, room temperature). The supernatants were discarded and the formazan crystals were dissolved with 200 μL of dimethyl sulfoxide. After 30 min of incubation at room temperature, absorbance was recorded at 570 nm, and the results were expressed as percentage of viable cells relative to the negative control. The cell viability percentage was used to calculate the toxin concentration that reduced cell viability by 50% (IC_50_), with the aid of the Calcusyn 2.1 software. The experiments were performed in independent triplicates.

### Quantification of intracellular ROS

Intracellular ROS generation was measured using 2',7'-dichlorodihydrofluorescein diacetate (H_2_DCFDA), as reported by Wang and Joseph [37]. HL-60, HL-60.Bcr-Abl, and HEK-293 cells (2×10^4^/well) were seeded in 96-well sterile black plates for 24 h, and treated with phosphate-buffered saline (PBS; negative control) or BmooLAAO-I at different concentrations, from 0.003075 to 0.038 μg/mL (HL-60 cells) or from 0.01225 to 0.192 μg/mL (HL-60.Bcr-Abl and HEK-293 cells). HL-60 and HL-60.Bcr-Abl cells were also treated with BmooLAAO-I in the presence or absence of 200 μg/mL catalase (Sigma-Aldrich, St. Louis, MO, USA). The supernatant was discarded, and the cells were washed with PBS and incubated with 100 μL of 10 μM H_2_DCFDA for 30 min, at 37 °C. The positive control cells were treated with 20 μL of 20 μM H_2_O_2_ for 20 min and washed with PBS. Finally, 100 μL of PBS were added to each well, and the fluorescence intensity was recorded in the fluorescent microplate reader Synergy H1, at the excitation and emission wavelengths of 485 and 528 nm, respectively. The percentage of intracellular ROS levels was normalized considering untreated cells (negative control) as 100%, and was calculated from the ratio between fluorescence intensity of each treated sample and fluorescence intensity of the negative control, multiplied by 100.

### Cell death quantification

HL-60, HL-60.Bcr-Abl, K562-S, and K562-R cells (1×10^5^) were seeded in 6-well plates and treated for 24 h with BmooLAAO-I at different concentrations: 0.003075-0.038, 0.01225-0.192, 0.01225-0.148, and 0.01225-0.166 μg/mL, respectively. Etoposide (VP-16) at 25 μM was used to induce cell death, as the positive control. Untreated cells were used as the negative control. The apoptosis levels were quantified using the annexin V-FITC/PI [38] and hypotonic fluorescent solution (HFS) methods [39].

Annexin V assay

After BmooLAAO-I treatment, cells were collected, centrifuged (240 ×*g*, 10 min, 4 °C), washed with 200 µL of annexin-binding buffer, and suspended in 100 µL of annexin V-FITC previously diluted 1:2,000 in annexin-binding buffer. After a 20-min incubation at room temperature, 1 µL of 250 µg/mL propidium iodide (PI) solution was added to each sample. Data from 10,000 events were analyzed in the FACSCanto flow cytometer (Becton-Dickinson, San Jose, CA, EUA) and the percentage of cells stained only with annexin V (annexin V^+^/PI^−^) or with both annexin V and PI (annexin V^+^/PI^+^) were quantified.

HFS assay

After BmooLAAO-I treatment, cells were collected, centrifuged (240 ×*g*, 10 min, 4 °C), and suspended in 400 µL of HFS (50 µg/mL PI in 0.1% sodium citrate and 0.1% Triton X-100). After incubation (20 min, 4 °C), data from 5,000 events were acquired and hipodiploid nuclei were quantified in the FACSCanto flow cytometer (Becton-Dickinson).

### Analysis of protein expression

HL-60, HL-60.Bcr-Abl, K562-S, and K562-R cells (1×10^6^) were seeded in 6-well plates and treated for 24 h with BmooLAAO-I at 0.003075-0.038, 0.01225-0.192, 0.01225-0.148, and 0.01225-0.166 μg/mL, respectively, or 25 μM VP-16 (positive control). Untreated cells were used as the negative control. Next, cells were collected and suspended in Western blotting lysis buffer (20 mM Tris-HCl pH 7.4, 150 mM NaCl, 1 mM EDTA, and phosphatase and protease inhibitors).

Total protein concentration was determined using the BCA Protein Assay Kit^®^ (Thermo Fischer Scientific), according to the manufacturer’s instructions. Equal protein amounts (20 μg) were submitted to SDS-PAGE analysis and transferred to polivinilidene fluoride membranes (Amersham ECL Plus^®^, GE Healthcare Life Sciences, Pittsburgh, PA, USA). The membranes were treated with blocking solution (5% non-fat dry milk, 0.01% sodium azide) for 2 h and incubated overnight at 18 °C with primary antibodies against caspase 3 (code #96625), caspase 8 (code #9746), caspase 9 (code #9502), PARP (code #9541, anti), and Bcl-2 (code #2870) (Cell Signaling Technology, Danvers, MA, USA), previously diluted in blocking solution to appropriate concentrations. After removing unbound primary antibodies, the membranes were washed with TBS-T buffer (100 mM Tris-HCl, 300 mM NaCl, 1 % Tween 20) and incubated (1 h, room temperature) with the respective peroxidase-conjugated anti-mouse or anti-rabbit secondary antibody (Sigma-Aldrich) diluted 1:2,000. The membranes were revealed by chemiluminescence according to the manufacturer’s instructions (Amersham ECL Plus^®^, GE Healthcare Life Sciences). The proteins β-tubulin (code #2146; Cell Signaling Technology), β-actin (code #A1978; Sigma-Aldrich), and γ-tubulin (code #T3320; Sigma-Aldrich) were used for sample loading normalization.

### DNA extraction and methylation pattern analysis

The methylation pattern was analyzed in K562-S and K562-R cells (5×10^6^ cells/well) treated for 24 h with BmooLAAO-I at 0.01225 and 0.0245 μg/mL in 6-well plates. Total DNA was extracted with QIAamp DNA Mini Kit and purified with minElute Reaction Cleanup kit (Qiagen Company, Hildren, Frankfurt, Germany). Total DNA concentration was determined using the NanoVue spectrophotometer (GE Healthcare Life Sciences). One μg of each sample was submitted to DNA restriction digestion using the EpiTect Methyl II DNA Restriction Kit (Qiagen Company). Next, the EpiTect Methyl PCR Arrays (Qiagen Company) was used to analyze methylation of the promoter region of the following apoptosis-related genes: *APAF1*, *BAD*, *BAG1*, *BAX*, *BCL2*, *BCL2L11*, *BCLAF1*, *BID*, *BIK*, *BIRC2*, *BNIP3L*, *CASP3*, *CASP9*, *CIDEB*, *CRADD*, *DAPK1*, *DFFA*, and *FADD.* Samples were analyzed by real-time PCR (StepOnePlus ™, Applied Biosystems) and the percentage of methylated and non-methylated genes were determined. The results were expressed as percentage of total input DNA (% of methylated DNA) associated with the corresponding heatmap created using the Hierarchical clustering method, with the aid of the Software MeV v4.8.1. All the assay kits were used according to the respective manufacturer’s instructions.

### RNA extraction, cDNA synthesis, and real-time PCR analysis

HL-60 cells (5×10^5^ cells/well) were treated with BmooLAAO-I at 0.003075 and 0.00615 μg/mL, while the Bcr-Abl^+^ cells HL-60.Bcr-Abl, K562-S, and K562-R cells (5×10^5^ cells/well) were treated with BmooLAAO-I at 0.01225 and 0.0245 μg/mL, for 24 h in 6-well plates. Untreated cells were used as the negative control. Total RNA was extracted using the Trizol^®^ method, following the manufacturer’s instructions (Invitrogen Life Technologies^®^, Carlsbad, USA). RNA concentrations were calculated from ratio of absorbance recorded at 260 nm and 280 nm (A260/A280), using the NanoVue spectrophotometer (GE Healthcare Life Sciences). Complementary DNA (cDNA) was reverse transcribed from 1 μg of the total RNA extracted, using the High Capacity cDNA reverse transcription^®^ kit (Applied Biosystems^®^, Foster City, EUA), according to the manufacturer’s recommendations. cDNA (diluted 1 to 4) samples were used to quantify gene expression (hypermethylated genes) by real-time PCR (StepOnePlus™ equipment, Applied Biosystems), using the assay kit SYBR Green PCR Master Mix (Applied Biosystems, Carlsbad, CA, USA) for the genes *BID* (BH3 interacting domain death agonist) and *FADD* (Fas associated via death domain) and the reference genes *Β-ACTIN* and *B2M*, and the assay kit TaqMan^®^ Gene Expression assays (Applied Biosystems, Foster City, EUA) for the gene *DFFA* (DNA fragmentation factor subunit alpha) and the reference genes *Β-ACTIN* and *GAPDH*. The Ct (*cycle threshold*) was used to calculate values of gene expression using the equation 2^-ΔΔCt^ (*fold change*). Three independent experiments were performed. The oligonucleotide sequences used for quantification of target gene expression are listed in Additional file 2.

### miRNA expression levels in leukemic cell lines

To analyze the level of apoptomiR expression in HL-60, HL-60.Bcr-Abl, K562-S, and K562-R cells, 2.5 ng of the total RNA extracted were used to synthesize cDNA, using the High Capacity cDNA reverse transcription^®^ assay kit (Applied Biosystems^®^), with specific loop-primers for each miRNA (Applied Biosystems^®^). cDNA (diluted 1:4) was used to detect expression of the apoptomiR miR-15a (predicted target gene *BCL2*), miR-16 (predicted target gene *BCL2*), and hsa-let-7d (predicted target gene *BCL2L1*). The expression levels of each miRNA were normalized using the reference miRNA RNU24 and RNU44. miRNA expression was quantified by real-time PCR (StepOnePlus™ equipment, Applied Biosystems), using the TaqMan^®^ microRNA assay kit for human samples (Applied Biosystems^®^, Foster City, USA). The Ct (*cycle threshold*) was used to calculate values of miRNA expression using the equation 2^-ΔΔCt^ (*fold change*).

### Data analysis

One-way Analysis of Variance (ANOVA) followed by the Tukey’s *post-hoc* test was used to compare the results from experimental groups (cell lines treated with BmooLAAO-I at different concentrations) and their respective negative control groups (cell lines not treated with the toxin), in the assays to determine ROS production, cytotoxicity, apoptosis, formation of hipodiploid nuclei, gene expression, and miRNA expression. The first four assays were carried out in three biological and technical replicates, while the last two assays were carried out in two biological and technical replicates. All the experimental data were analyzed using the GraphPad Prism version 5.0 software (GraphPad Software, San Diego, California, USA), with values of p<0.05 considered as significant. The power of the statistical test applied for each cell line and assay was determined by one-way ANOVA with 5% of significance. All the tests had power greater than 0.962.

## Results

### Isolation and purification of BmooLAAO-I

BmooLAAO-I was successfully purified through three chromatographic steps. After the first purification step on a CM-Sepharose column, the LAAO-active fraction was identified using enzymatic assays (Additional file 3A). The resulting fraction was concentrated and subsequently applied onto a Phenyl-Sepharose CL-4B column (Additional file 3B). The resulting LAAO-active fraction was concentrated and applied onto a Benzamidine Sepharose affinity column (Additional file 3C). An LAAO-active fraction was obtained with high purity, as analyzed by C18 reversed-phase HPLC (Additional file 3D) and SDS-PAGE (Additional file 3D, inset).

The N-terminal amino acid sequence of the isolated protein obtained by automatic Edman degradation resulted in a sequence of 35 amino acid residues (ADDRNPLEECFRETDYEEFLETAKNGLSTTSNKKL) with high identity (95%) to the N-terminal sequence of the BmooLAAO-I isolated from *B. moojeni* venom by Stábeli et al. [31].

### BmooLAAO-I selective cytotoxicity to leukemic cell lines is mediated by H_2_O_2_ release

**Figure 1.**
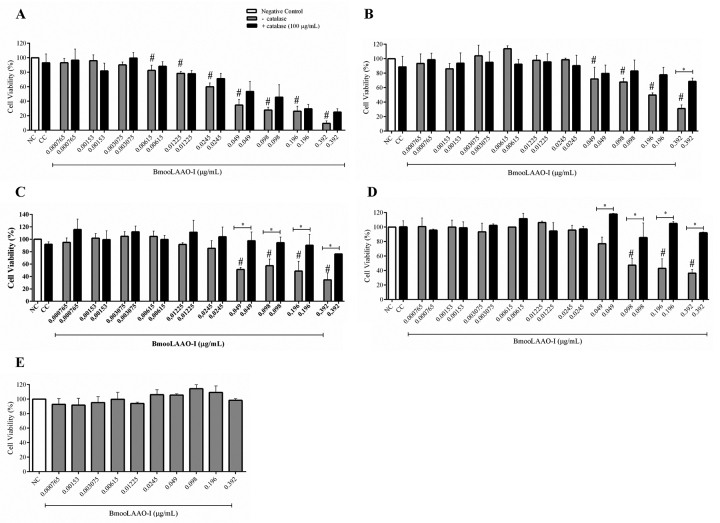
Cytotoxicity of BmooLAAO-I towards four tumor cell lines. **(A)** HL-60, **(B)** HL-60.Bcr-Abl, **(C)** K562-S, and **(D)** K562-R cells were treated with the toxin for 24 h, in the presence or absence of 100 μg/mL of catalase. **(E)** HEK-293 cells were treated with the toxin for 24 h only without catalase due to the lack of cytotoxicity of BmooLAAO-I towards this cell line. Results are expressed as mean ± standard deviation of the percentage of cell viability from three independent experiments assayed in triplicate. NC: negative control (untreated cells); CC: catalase control (cells treated only with catalase). ^#^ *p* < 0.05 *vs.* NC; *p < 0.05 *vs.* catalase (−) (one-way ANOVA combined with the Tukey’s *post-hoc* test).

We assessed the BmooLAAO-I cytotoxicity to the tumor cell lines HL-60, HL-60.Bcr-Abl, K562-S, and K562-R, as well as to the non-tumor cell line HEK-293 (Fig. 1) and PBMC (Additional file 4). The toxin decreased cell viability of HL-60 cells at concentrations greater than 0.00615 μg/mL, in a concentration-dependent manner, reducing cell viability to 9% at the highest concentration tested (0.392 μg/mL, Fig. 1A) and yielding IC_50_ = 0.038 μg/mL. BmooLAAO-I at the highest concentration tested (0.392 μg/mL) strongly reduced cell viability to around 30% in HL-60.Bcr-Abl, K562-S, and K562-R cells, and afforded IC_50_ values of 0.192, 0.148, and 0.166 μg/mL, respectively (Fig. 1B-D). The toxin significantly reduced cell viability of HL-60.Bcr-Abl and K562-S cells at concentrations greater than 0.049 μg/mL, and of K562-R cells at concentrations greater than 0.098 μg/mL. Compared with untreated cells, treatment with BmooLAAO-I did not significantly alter the cell viability (90-100%) of HEK-293 cells (Fig. 1E) and PBMC (Additional file 4), under the conditions assessed.

To address whether BmooLAAO-I cytotoxicity was associated with H_2_O_2_ release, we analyzed cell viability in the presence of catalase, a H_2_O_2_-degrading enzyme. Catalase at 100 μg/mL increased cell viability of all leukemic cell lines, and exerted stronger effects in HL-60.Bcr-Abl, K562-S, and K562-R treated with the highest toxin concentration (0.392 μg/mL), in which catalase augmented cell viability by 30 to 50% (Fig. 1B- D).

Each cell line was then treated with three BmooLAAO-I concentrations - the two lowest concentrations that reduced cell viability and the IC_50_ value - in the presence or not of catalase at 200-400 μg/mL (Additional file 5). The three catalase concentrations tested increased cell viability to the same extent as the previously tested concentration of 100 μg/mL, in all tumor cell lines. Catalase significantly augmented cell viability of HL-60 and HL-60.Bcr-Abl cells treated with the two highest toxin concentrations (Additional file 5A- B), and of K562-S and K562-R cells treated with the three toxin concentrations tested (Additional file 5C- D).

### BmooLAAO-I increases ROS generation in leukemic cell lines

**Figure 2.**
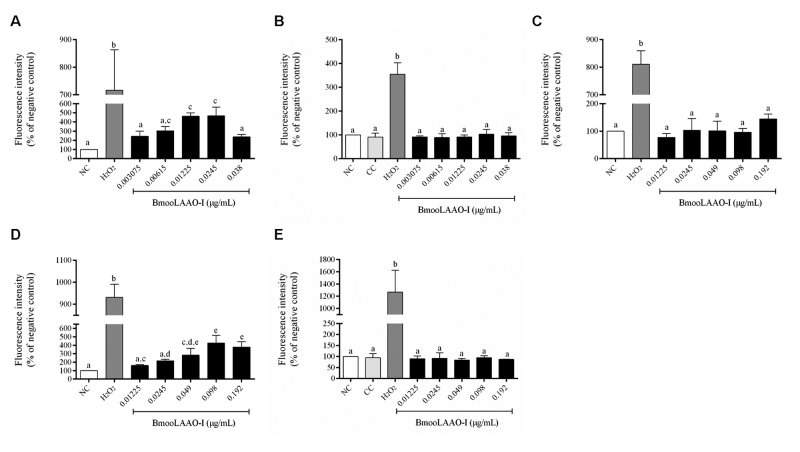
Intracellular ROS generation in cells treated with BmooLAAO-I for 24 h. **(A**, **B)** Fluorescence intensity of H_2_DCFDA was measured in HL-60, **(C)** HEK-293 and **(D**, **E)** HL-60.Bcr-Abl cells. HL-60 and HL-60.Bcr-Abl cells were treated with the toxin in **(A**, **D)** the absence or **(B**, **E)** presence of 200 μg/mL of catalase. Results are expressed as mean ± standard deviation of three independent experiments. NC: negative control (untreated cells); CC: catalase control (cells treated only with catalase). Values not sharing the same letter are significantly different from each other (*p* < 0.05; one-way ANOVA combined with the Tukey’s *post-hoc* test).

To confirm that BmooLAAO-I cytotoxicity resulted from ROS production, we quantified intracellular ROS generation in the leukemic cell lines HL-60 and HL-60.Bcr-Abl, and in the non-tumor cell line HEK-293 (Fig. 2). BmooLAAO-I at intermediate concentrations (0.01225 and 0.0245 µg/mL) enhanced ROS levels in HL-60 cells (Fig. 2A) and, at concentrations greater than 0.049 µg/mL, it enhanced ROS levels in HL-60.Bcr-Abl cells (Fig. 2D). The toxin did not alter ROS levels in HEK-293 cells (Fig. 2C). Catalase mitigated the toxin-elicited increased ROS levels in HL-60 and HL-60.Bcr-Abl cells (Fig. 2B, 2E). These results indicate the association between ROS production and BmooLAAO-I cytotoxicity in these leukemic cell lines.

### BmooLAAO-I induces apoptosis in leukemic cell lines

**Figure 3.**
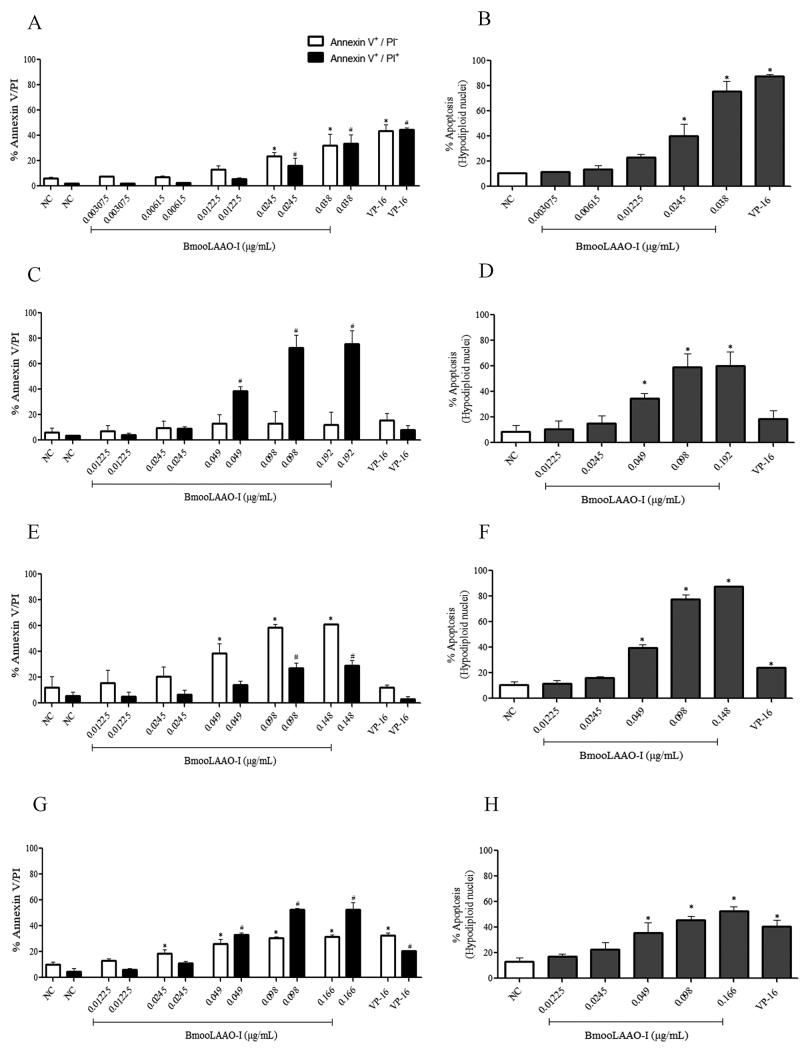
Quantification of apoptosis levels induced by BmooLAAO-I. The percentage of annexin V^+^/PI^-^ (white bars) and annexin V^+^/PI^+^ (black bars) was determined in **(A)** HL-60, **(C)** HL-60.Bcr-Abl, **(E)** K562-S, and **(G)** K562-R cells. The percentage of hipodiploid nuclei was determined in **(B)** HL-60, **(D)** HL-60.Bcr-Ab, **(F)** K562-S, and **(H)** K562-R cells, using the hypotonic fluorescent solution method. Results are expressed as mean ± standard deviation of three independent experiments. NC: negative control (untreated cells); VP-16: etoposide. *p < 0.05 *vs.* NC (annexin V^+^/PI^-^ and hypodiploid nuclei); ^#^ *p* < 0.05 *vs.* NC (annexin V^+^/PI^+^) (one-way ANOVA combined with the Tukey’s *post-hoc* test).

Considering the cytotoxic effect of BmooLAAO-I in leukemic cell lines, we investigated whether BmooLAAO-I induced apoptosis in these tumor cells (Fig. 3). BmooLAAO-I at the concentration of 0.0245 and 0.038 µg/mL increased the percentage of annexin V^+^/PI^-^ cells (from 23 to 32%), annexin V^+^/PI^+^ cells (from 16 to 33%), and hipodiploid nuclei (from 40 to 76%) in HL-60 cells (Fig. 3A-B). The toxin at the concentration range of 0.049-0.192 µg/mL augmented the percentage of annexin V^+^/PI^+^ cells (38-75%) and hipodiploid nuclei (34-70%), but not of annexin V^+^/PI^-^ cells in HL-60.Bcr-Abl cells (Fig. 3C- D).

In K562-S cells, the toxin increased the percentage of annexin V^+^/PI^-^ cells (35-61%) and hipodiploid nuclei (40-87%) at 0.049-0.148 µg/mL, and the percentage of annexin V^+^/PI^+^ cells (26-28%) at 0.098 and 0.148 µg/mL (Fig. 3E- F). In K562-R cells, the toxin augmented the percentage of annexin V^+^/PI^-^ cells (18-31%) at 0.0245-0.166 µg/mL, and the percentage of annexin V^+^/PI^+^ cells (33-52%) and hipodiploid nuclei (35-52%) at 0.049-0.166 µg/mL (Fig. 3G-H). The toxin at 0.098 and 0.166 µg/mL induced weaker formation of hipodiploid nuclei in K562-R cells than in K562-S cells (Additional file 6).

VP16 induced cell apoptosis in HL-60 and K562-R cells, but not in HL-60.Bcr-Abl and K562-S cells (Fig. 3). This compound induced the formation of hypodiploid nuclei with strong intensity in HL-60 cells (87%, Fig. 3B), moderate intensity in K562-R cells (40%, Fig. 3H), and weak intensity in K562-S (24%, Fig. 3F) and HL-60.Bcrl-Abl (18%, Fig. 3D) cells.

### BmooLAAO-I activates caspases 3, 8 and 9 in leukemic cells

**Figure 4.**
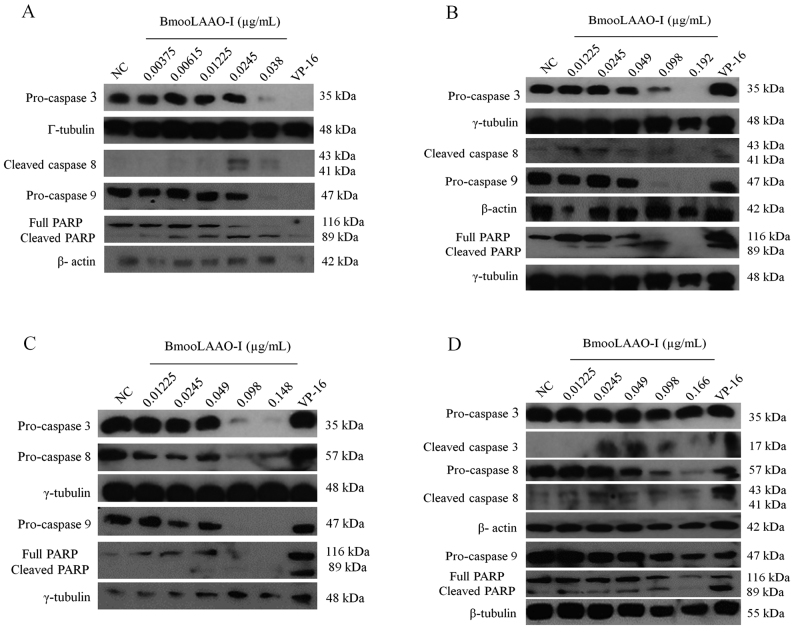
Western blotting analysis of BmooLAAO-I-induced expression of cleaved caspases in tumor cell lines. **(A)** The levels of caspases 3, 8 and 9 were determined in HL-60, **(B)** HL-60.Bcr-Abl, **(C)** K562-S, and **(D)** K562-R cells after 24 h of treatment with BmooLAAO-I. Decreased pro-caspase expression and increased expression of active (cleaved) forms indicates caspase activation. NC: negative control (untreated cells). VP-16: etoposide (positive control).

To examine whether BmooLAAO-I induced apoptosis via the intrinsic or extrinsic pathway activation, we analyzed the expression levels of caspases 3, 8, and 9. The toxin lowered expression of pro-caspases 3, 8, and 9 in all tumor cell lines (Fig. 4) and increased the levels of cleaved caspase 8 in K562-R cells (Fig. 4D) and HL-60.Bcr-Abl cells (Fig. 4B). 

### BmooLAAO-I modulates the methylation pattern of apoptosis-related genes in K562-S cells

**Figure 5.**
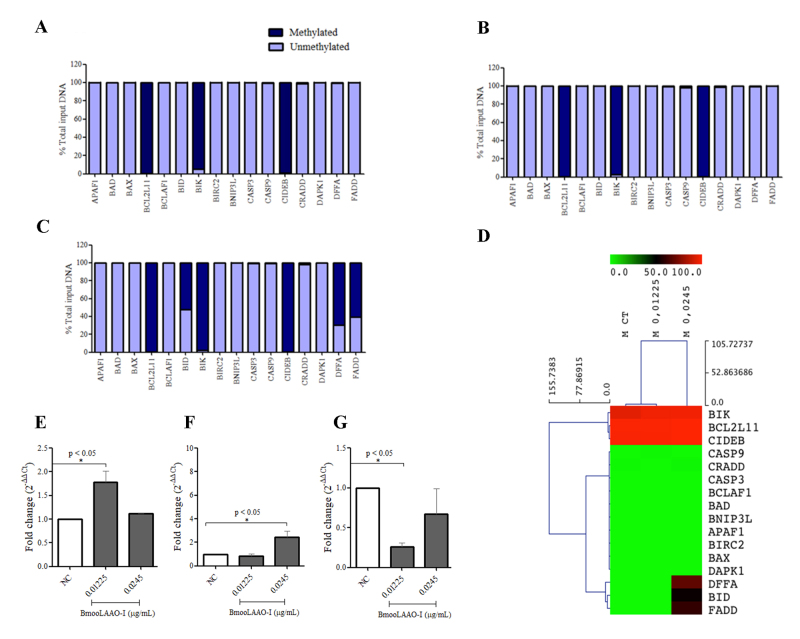
BmooLAAO-I modulates the apoptosis-related gene methylation pattern in K562-S cells. The percentage of methylation of the promoter region of apoptosis-related genes was quantified by real-time PCR in cells treated with BmooLAAO-I for 24 h. **(A)** Untreated cells (negative control). **(B)** Cells treated with the toxin at 0.01225 µg/mL. **(C)** Cells treated with the toxin at 0.0245 µg/mL. **(D)** Heatmap of sample clustering according to the percentage of methylated DNA. The horizontal bar in the top of the heatmap represents the color scale of percentage of methylation ranging from 0-100%. Expression levels of hypermethylated genes in K562-S cells treated with BmooLAAO-I. Expression of the genes **(E)** *BID*, **(F)** *FADD*, and **(G)** *DFFA* was quantified by real-time PCR after a 24 h treatment with BmooLAAO-I at sublethal concentrations (0.01225 and 0.0245 µg/mL). Results were expressed as mean fold change ± standard deviation of three independent experiments. NC: negative control (untreated cells). *p < 0.05 *vs.* NC (one-way ANOVA followed by the Tukey’s *post-hoc* test).

Considering the mechanisms by which BmooLAAO-I induced apoptosis in leukemic cells, we examined whether it modulated the DNA methylation pattern in the promoter region of apoptosis-related genes in K562-S (Fig. 5) and K562-R (Additional file 7) cells. The cells were treated with BmooLAAO-I at the sublethal concentrations of 0.01225 and 0.0245 µg/mL. Compared with the negative control (Fig. 5A), the pro-apoptotic genes *BID*, *FADD*, and *DFFA* were hypermethylated (52.42%, 60.45%, and 68.51%, respectively) in K562-S cells treated with the toxin at 0.0245 µg/mL (Fig. 5C). Heatmap analysis showed that the three genes were hypermethylated, but the other apoptosis-related-genes exhibited methylation levels similar to those detected in the negative control (Fig. 5D). The toxin did not alter the methylation pattern of apoptosis-related genes in K562-R cells (Additional file 7).

### BmooLAAO-I modulates expression of BID, FADD, and DFFA genes

Since BmooLAAO-I promoted hypermethylation of the genes *BID, FADD*, and *DFFA*, we analyzed their expression levels in K562-S cells (Fig. 5E-G). The toxin at 0.01225 µg/mL upregulated *BID* expression [fold change (fc) = 1.78, Fig. 5E] and downregulated *DFFA* expression (fc = 0.26, Fig. 5G). At the concentration of 0.0245 µg/mL, the toxin did not alter the *BID* and *DFFA* expression levels, but it upregulated *FADD* expression (fc = 2.42, Fig. 5F).

### BmooLAAO-I modulates miR-16 and Bcl-2 protein expression in K562-R cells

**Figure 6.**
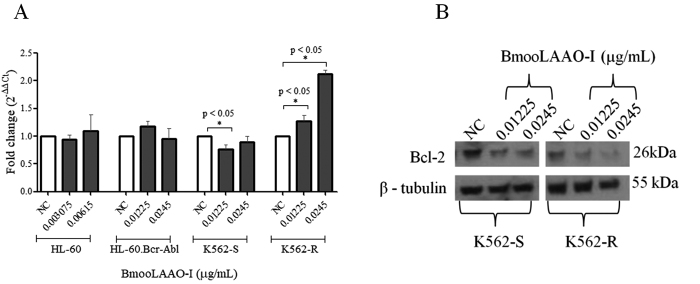
Quantification of miR-16 and Bcl-2 expression in tumor cell lines treated with BmooLAAO-I. The expression levels of **(A)** the apoptomiR miR-16 and **(B)** Bcl-2 were quantified by real-time PCR in HL-60, HL-60.Bcr-Abl, K562-S, and K562-R cells treated for 24 h with BmooLAAO-I at sublethal concentrations. Results were expressed as mean fold change ± standard deviation of three independent experiments. NC: negative control (untreated cells). *p < 0.05 *vs.* NC (one-way ANOVA followed by the Tukey’s *post-hoc* test).

Considering that regulation by miRNA is another important epigenetic mechanism involved with tumorigenesis and apoptosis resistance, we detected the expression levels of the apoptomiR miR-15a, miR-16, and hsa-let-7d in leukemic cell lines treated with BmooLAAO-I at sublethal concentrations. BmooLAAO-I did not alter the expression levels of miR-15a (Additional file 8A) and hsa-let-7d (Additional file 8B). Notably, the toxin downregulated miR-16 expression in K562-S cells at the concentration of 0.01225 µg/mL (fc = 0.75), and upregulated miR-16 expression in K562-R cells at the concentrations of 0.01225 and 0.0245 µg/mL (fc = 1.26 and 2.12, respectively) (Fig. 6A). The expression level of the Bcl-2 protein, one of the miR-15a/miR-16 predicted target gene, decreased in K562-S and K562-R cells treated with both toxin concentrations (Fig. 6B).

## Discussion

Natural compounds, including SV-LAAO, have exhibited strong antitumor activity [22,28-30,32,40]. In the present study, BmooLAAO-I decreased cell viability of all the leukemic cell lines tested: HL-60, HL-60.Bcr-Abl, K562-S, and K562-R. To address whether H_2_O_2_ released during the LAAO enzymatic activity of BmooLAAO-I mediated cell death, we carried out the cytotoxicity assay in the presence of 100 µg/mL catalase. Addition of such H_2_O_2_-degrading enzyme increased cell viability of the four leukemic cell lines treated with different toxin concentrations. This finding is in line with our previous reports that 100 µg/mL catalase mitigates cytotoxicity of CR-LAAO towards HL-60, HL-60.Bcr-Abl, K562, and KCL22 cells [30], as well as with other literature reports that catalase mitigates cytotoxicity of LAAO from *B. pirajai*, *Bothrops atrox*, *Bothrops pauloensis*, *Lachesis muta*, and *Ophiophagus hannah* to the tumor cell lines SBKR-3, Jurkat, EAT, MCF-7, A549, Jurkat, and B16/F10 [22,35,36,41,42].

One essential mechanism for BmooLAAO-I cytotoxicity is ROS overproduction. We confirmed that BmooLAAO-I induced ROS production in leukemic cell lines in a concentration-dependent manner, but it did not alter ROS levels in the non-tumor HEK cells. Catalase not only mitigated the toxin-mediated ROS overproduction due to degradation of H_2_O_2_ but also increased cell viability, indicating that H_2_O_2_ was a key player on the BmooLAAO-I cytotoxicity to leukemic cell lines, especially to Bcr-Abl^+^ cells. In agreement with our findings, LAAO isolated from *Bothrops alternatus* and *Bothrops jararacussu* snake venoms induce ROS overproduction in many tumor cell types, including hepatocarcinoma, leukemia T, breast cancer, adenocarcinoma, and melanoma; however, the enhanced ROS levels are associated with anti-tumor effects due their contribution to cell death and DNA damage [43,44].

Considering the strong pharmacological effects and the risk of toxicity of snake venom toxins to humans, their cytotoxicity must be evaluated in non-tumor cells. This study demonstrated that BmooLAAO-I was not cytotoxic to the non-tumor cell line HEK-293 and to PBMC cells, corroborating previous reports that SV-LAAO is more cytotoxic to tumor cells than to non-tumor cells [28,29,35,45]. 

After analyzing the BmooLAAO-I-induced cytotoxicity and ROS production, we examined the toxin ability to sensitize and/or induce apoptosis in leukemic and non-leukemic cells. Apoptosis is an important cell process to be analyzed during antitumor drug development, because it is one of the main routes of clearance of neoplastic cells [46-48]. It is well-known that Bcr-Abl^+^ cells are more resistant to apoptosis induced by chemotherapeutic agents and classical apoptogenic stimuli [49,50]. Hence, our finding that BmooLAAO-I was capable of inducing apoptosis in both Bcr-Abl^+^ and Bcr-Abl^-^ leukemic cells is relevant. The fact that K562-S cells are more sensitive to the TKI imatinib mesylate than K562-R cells [51,52] may explain, at least in part, why this cell was more sensitive to BmooLAAO-I cytotoxicity. 

The glycan moiety of SV-LAAO favors their anchoring in tumor cells and increases the local concentration of H_2_O_2_, which thereby causes oxidative damage and induces apoptosis [53]. Many studies have demonstrated the apoptosis-inducing potential of SV-LAAO in a variety of tumor cell lines, such as HL-60, A2780, K562, Jurkat, B16/F10, and A549 [20,22,23,25,54-56]. Our research team has reported that BpirLAAO and CR-LAAO stimulate apoptosis in primary cells from chronic myeloid leukemia (CML) patients and in Bcr-Abl^+^ cell lines [28,29]. 

Apoptosis stimulation by BmooLAAO-I is accompanied by activation of caspases 3, 8, and 9 in the tumor cell lines, indicating that this toxin induces apoptosis via the intrinsic and extrinsic pathways. This finding is in line with previous reports from our research team that BpirLAAO-I, CR-LAAO, and BjussuLAAO-II (a LAAO isolated from *B. jararacussu* venom) promote activation of caspases 3, 8 and 9 in tumor cell lines [23,28,30,33]. Many authors have reported the potential of SV-LAAO to activate caspases in tumor cell lines, including HeLa, A549, and MCF-7 cells [36,57,58].

The deregulated expression of a number of apoptosis-related genes in CML patients may be explained, at least in part, by epigenetic deregulation such as DNA methylation and altered miRNA expression [6,17]. To better understand the molecular mechanisms by which the toxin induced apoptosis, we examined whether BmooLAAO-I modulated the DNA methylation pattern of apoptosis-related genes, as well as the expression levels of apoptomiR. 

In K562-S cells, the toxin at 0.0245 µg/mL induced hypermethylation of the promoter region of the genes *BID*, *FADD*, and *DFFA*, but it did not supress their gene expression levels. Paradoxically, the toxin increased expression of the pro-apoptotic genes *BID* and *FADD* at 0.01225 and 0.0245 µg/mL, respectively, and decreased *DFFA* expression at 0.01225 µg/mL; hence, the hypermethylation level was not sufficient to suppress *BID* and *FADD* gene expression in cells treated with 0.0245 µg/mL BmooLAAO-I. Essentially, DNA methylation does not imply gene silencing because suppression of gene expression requires recruitment of regulatory proteins and chromatin remodeling factors to the methylated region. However, DNA methylation is the key step of these gene regulation [59]. We should also consider that other epigenetic mechanisms can regulate gene expression, such as miRNA expression and histone acetylation [60]. Additional studies are required to better elucidate the role that DNA methylation plays on the BmooLAAO-I-induced expression of apoptosis genes. 

The BmooLAAO-I-induced upregulation of *BID* and *FADD* gene expression suggests that the toxin sensitizes leukemic cells to apoptosis, which is an advantage for the treatment of neoplasias, including CML [61]. During apoptosis activation, *FADD* is recruited with pro-caspase 8 and activates caspase 8, which in turn activates caspase 3. The protein Bid represents a connection point between the extrinsic and intrinsic apoptosis pathways, and it is activated by caspase 8-mediated cleavage [62,63]. *DFFA* plays a controversial role on apoptosis induction because it can act as an anti- or pro-apoptotic gene [64]. 

Analysis of miRNA expression pattern in some type of tumors can explain or elucidate the mechanisms involved in tumor resistance or sensitivity to different treatments, and help to select therapeutic approaches [65,66]. We analyzed the apoptomiR expression profile in K562-S and K562-R cells. The major finding was the elevated expression of the apoptomiR miR-16 in K562-R cells treated with BmooLAAO-I. The increased expression of miR-16 is associated with lowered levels of the anti-apoptotic protein Bcl-2 [17-19]. BmooLAAO-I reduced Bcl-2 levels in K562-R cells, indicating that the expression levels of miR-16 and Bcl-2 were inversely correlated. Upregulation of miR-16 expression by BmooLAAO-I is of great importance because it lowers the levels of Bcl-2 and increases the sensitivity of Bcr-Abl^+^ leukemic cells to apoptosis. The Bcr-Abl tyrosine kinase activity is associated with apoptosis inhibition mediated by increased levels of anti-apoptotic proteins [49,67]. 

## Conclusion

Taken together, the findings of the present study demonstrate the antitumor potential of BmooLAAO-I, which acts through ROS production, apoptosis induction, and upregulation of expression of the apoptomiR miR-16. In addition, the selective toxicity towards tumor cells and the low toxicity to non-tumor cells and PBMC cells open new perspectives for application of BmooLAAO-I in antitumor therapy.

### Abbreviations

ANOVA: analysis of variance; apoptomiR: microRNA that regulate apoptosis-related genes; BmooLAAO: L-amino acid oxidase from *Bothrops moojeni*; BpirLAAO: L-amino acid oxidase from *Bothrops pirajai*; cDNA: complementary DNA; CML: chronic myeloid leukemia; CR-LAAO: L-amino acid oxidase from *Calloselasma rhodostoma*; H_2_DCFDA: 2',7'-dichlorodihydrofluorescein diacetate; HFS: hypotonic fluorescent solution; LAAO: L-amino acid oxidase; miRNA: microRNA; MTT: 3-(4,5-dimethylthiazol-2-yl)-2,5-diphenyltetrazolium bromide; PBMC: peripheral blood mononuclear cells; ROS: reactive oxygen species; SV-LAAO: L-amino acid oxidase from snake venom; TKI: tyrosine kinase inhibitor; VP-16: etoposide.

## Supplementary material

The following online material is available for this article:

Additional file 1. Parameters of L-amino acid oxidase from *B. moojeni* snake venom purification procedure.

Additional file 2. Oligonucleotide sequences used for quantification of target gene expression.

Additional file 3. Purification of the L-amino acid oxidase BmooLAAO-I from *Bothrops moojeni* snake venom. **(A)** Chromatographic profile of 200 mg of desiccated venom applied onto a CM-sepharose column previously equilibrated with 0.05 M NH_4_NaHCO_3_ buffer, pH 8.0, and eluted with a concentration gradient of up to 1.0 M of the same buffer (buffer B) at room temperature. The LAAO-active fraction eluted in the first peak. **(B)** Chromatographic profile of the pooled and concentrated LAAO-active fraction obtained in (A), applied onto a phenyl-sepharose column previously equilibrated with 0.02 M Tris-HCl buffer, pH 7.6 (buffer B) containing 4 M NaCl, and eluted with a discontinuous gradient of 4-0 M NaCl in the same buffer. **(C)** Chromatographic profile of the pooled and concentrated LAAO-active fraction obtained in (B), applied onto a benzamidine sepharose column pre-equilibrated with 20 mM Tris-HCl, pH 7.4 (buffer A), and eluted with a step gradient of 20 mM Tris-HCl containing 1.0 M NaCl, pH 7.4 (buffer B). **(D)** Purity of the active enzyme BmooLAAO-I assessed by SDS-PAGE (inset) and reversed-phase HPLC.

Additional file 4. Cytotoxicity of BmooLAAO-I towards PBMC, at 24 h post-treatment. Results are expressed as mean ± standard deviation of the percentage of cell viability from three samples assayed in triplicate. NC: negative control (untreated cells). *p* > 0.05 *vs*. NC (one-way ANOVA combined with the Tukey’s *post-hoc* test).

Additional file 5. Cytotoxicity of BmooLAAO-I towards tumor cell lines in the presence of 200-400 μg/mL of catalase. **(A)** HL-60 cells, **(B)** HL-60.Bcr-Abl cells, **(C)** K562-S cells, and **(D)** K562-R cells. Results are expressed as mean ± standard deviation of the percentage of cell viability from three independent experiments assayed in triplicate. Cells were treated with the toxin for 24 h. NC: negative control (untreated cells); CC: catalase control (cells treated with catalase only). **p* < 0.05 *vs.* (−) catalase (one-way ANOVA combined with the Tukey’s *post-hoc* test).

Additional file 6. Comparison between BmooLAAO-I-induced apoptosis levels in K562-S and K562-R cells. **(A)** Total percentage of annexin-V-stained cells. **(B)** Percentage of hipodiploid nuclei. Results are expressed as mean ± standard deviation of three independent experiments. NC: negative control (untreated cells). *p < 0.05 *vs.* NC (one-way ANOVA combined with the Tukey’s *post-hoc* test).

Additional file 7. BmooLAAO-I did not alter the methylation pattern of apoptosis-related genes in K562-R cells. The percentage of methylation of the promoter region of apoptosis-related genes was quantified by real-time PCR in cells treated with BmooLAAO-I for 24 h. **(A)** Untreated cells (negative control). **(B)** Cells treated with the toxin at 0.01225 µg/mL. **(C)** Cells treated with the toxin at 0.0245 µg/mL. **(D)** Heatmap of sample clustering according to the percentage of methylated DNA. The horizontal bar in the top of the heatmap represents the color scale of percentage of methylation ranging from 0-100%.

Additional file 8. ApoptomiRs expression in tumor cell lines treated with BmooLAAO-I. Real-time PCR quantification of the apoptomiRs **(A)** miR-15a and **(B)** has-let-7d in HL-60, HL-60.Bcr-Abl, K562-S, and K562-R cells treated for 24 h with BmooLAAO-I at sublethal concentrations. Results are expressed as mean fold change ± standard deviation of three independent experiments. NC: negative control (untreated cells). **p* < 0.05 *vs.* NC (one-way ANOVA followed by the Tukey’s *post-hoc* test).

## References

[B1] Vaidya S, Ghosh K, Vundinti BR (2011). Recent developments in drug resistance mechanism in chronic
myeloid leukemia: a review. Eur J Haematol.

[B2] de Castro Sant’ Anna C, Ferreira AG, Soares P, Tuji F, Paschoal E, Chaves LC (2018). Molecular biology as a tool for the treatment of
cancer. Clin Exp Med.

[B3] Breccia M, Alimena G (2014). Second-generation tyrosine kinase inhibitors (Tki) as salvage
therapy for resistant or intolerant patients to prior TKIs. Mediterr J Hematol Infect Dis.

[B4] Chereda B, Melo JV (2015). Natural course and biology of CML. Ann Hematol.

[B5] Patel AB, O’Hare T, Deininger MW (2017). Mechanisms of resistance to ABL kinase inhibition in chronic
myeloid leukemia and the development of next generation ABL kinase
inhibitors. Hematol Oncol Clin North Am.

[B6] Koschmieder S, Vetrie D (2018). Epigenetic dysregulation in chronic myeloid leukaemia: A myriad
of mechanisms and therapeutic options. Semin Cancer Biol.

[B7] Stahl M, Kohrman N, Gore SD, Kim TK, Zeidan AM, Prebet T (2016). Epigenetics in cancer: A hematological
perspective. PLoS Genet.

[B8] You RI, Ho CL, Hung HM, Hsieh YF, Ju JC, Chao TY (2012). Identification of DNA methylation biomarkers in
imatinib-resistant chronic myeloid leukemia cells. Gen Med Biomark Health Sci.

[B9] Esteller M (2008). Epigenetics in cancer. N Engl J Med.

[B10] Wang S, Wu W, Claret FX (2017). Mutual regulation of microRNAs and DNA methylation in human
cancers. Epigenetics.

[B11] Meng H, Cao Y, Qin J, Song X, Zhang Q, Shi Y (2015). DNA methylation, its mediators and genome
integrity. Int J Biol Sci.

[B12] Chim CS, Wong KY, Qi Y, Loong F, Lam WL, Wong LG (2010). Epigenetic inactivation of the miR-34a in hematological
malignancies. Carcinogenesis.

[B13] Piletič K, Kunej T (2016). MicroRNA epigenetic signatures in human disease. Arch Toxicol.

[B14] Venturini L, Battmer K, Castoldi M, Schultheis B, Hochhaus A, Muckenthaler MU (2007). Expression of the miR-17-92 polycistron in chronic myeloid
leukemia (CML) CD34+ cells. Blood.

[B15] Zhao H, Wang D, Du W, Gu D, Yang R (2010). MicroRNA and leukemia: tiny molecule, great
function. Crit Rev Oncol Hematol.

[B16] Gordon JEA, Wong JJL, Rasko JEJ (2013). MicroRNAs in myeloid malignancies. Br J Haematol.

[B17] Ferreira AF, Moura LG, Tojal I, Ambrósio L, Pinto-Simões B, Hamerschlak N (2014). ApoptomiRs expression modulated by BCR-ABL is linked to CML
progression and imatinib resistance. Blood Cells Mol Dis.

[B18] Calin GA, Dumitru CD, Shimizu M, Bichi R, Zupo S, Noch E (2002). Nonlinear partial differential equations and applications:
Frequent deletions and down-regulation of micro- RNA genes miR15 and miR16
at 13q14 in chronic lymphocytic leukemia. Proc Nat Acad Sci.

[B19] Cimmino A, Calin GA, Fabbri M, Iorio MV, Ferracin M, Shimizu M (2005). miR-15 and miR-16 induce apoptosis by targeting
BCL2. Proc Nat Acad Sci.

[B20] Torii S, Naito M, Tsuruo T (1997). Apoxin I, a novel apoptosis-inducing factor with L-amino acid
oxidase activity purified from Western diamondback rattlesnake
venom. J Biol Chem.

[B21] Guo C, Liu S, Yao Y, Zhang Q, Sun MZ (2012). Past decade study of snake venom L-amino acid
oxidase. Toxicon.

[B22] de Melo Alves Paiva R, de Freitas Figueiredo R, Antonucci GA, Paiva HH, de Lourdes Pires Bianchi M, Rodrigues KC (2011). Cell cycle arrest evidence, parasiticidal and bactericidal
properties induced by l-amino acid oxidase from Bothrops atrox snake
venom. Biochimie.

[B23] Costa TR, Menaldo DL, Zoccal KF, Burin SM, Aissa AF, Castro FA de (2017). CR-LAAO, an L-amino acid oxidase from Calloselasma rhodostoma
venom, as a potential tool for developing novel immunotherapeutic strategies
against cancer. Sci Rep.

[B24] Suhr SM, Kim DS (1996). Identification of the snake venom substance that induces
apoptosis. Biochem Biophys Res Commun.

[B25] Souza DH, Eugenio LM, Fletcher JE, Jiang MS, Garratt RC, Oliva G (1999). Isolation and structural characterization of a cytotoxic L-amino
acid oxidase from Agkistrodon contortrix laticinctus snake venom:
preliminary crystallographic data. Arch Biochem Biophys.

[B26] Zhang L, Wu WT (2008). Isolation and characterization of ACTX-6: a cytotoxic L-amino
acid oxidase from Agkistrodon acutus snake venom. Nat Prod Res.

[B27] Fung SY, Lee ML, Tan NH (2015). Molecular mechanism of cell death induced by king cobra
(Ophiophagus hannah) venom l-amino acid oxidase. Toxicon.

[B28] Burin SM, Ayres LR, Neves RP, Ambrósio L, de Morais FR, Dias-Baruffi M (2013). L-amino acid oxidase isolated from Bothrops pirajai induces
apoptosis in BCR-ABL-positive cells and potentiates imatinib mesylate
effect. Basic Clin Pharmacol Toxicol.

[B29] Burin SM, Ghisla S, Ouchida AT, Aissa AF, Coelho MGB, Costa TR (2016). CR-LAAO antileukemic effect against Bcr-Abl(+) cells is mediated
by apoptosis and hydrogen peroxide. Int J Biol Macromol.

[B30] Burin SM, Berzoti-Coelho MG, Cominal JG, Ambrosio L, Torqueti MR, Sampaio SV (2016). The L-amino acid oxidase from Calloselasma rhodostoma snake venom
modulates apoptomiRs expression in Bcr-Abl-positive cell
lines. Toxicon.

[B31] Stábeli RG, Sant’Ana CD, Ribeiro PH, Costa TR, Ticli FK, Pires MG (2007). Cytotoxic L-amino acid oxidase from Bothrops moojeni: biochemical
and functional characterization. Int J Biol Macromol.

[B32] Costa TR, Carone SEI, Tucci LFF, Menaldo DL, Rosa-Garzon NG, Cabral H (2018). Kinetic investigations and stability studies of two Bothrops
L-amino acid oxidases. J Venom Anim Toxins Incl Trop Dis.

[B33] Carone SEI, Costa TR, Burin SM, Cintra ACO, Zoccal KF, Bianchini FJ (2017). A new l-amino acid oxidase from Bothrops jararacussu snake venom:
Isolation, partial characterization, and assessment of pro-apoptotic and
antiprotozoal activities. Int J Biol Macromol.

[B34] Mosmann T (1983). Rapid colorimetric assay for cellular growth and survival:
application to proliferation and cytotoxicity assays. J Immunol Methods.

[B35] Izidoro LFM, Ribeiro MC, Souza GRL, Sant’Ana CD, Hamaguchi A, Homsi-Brandeburgo MI (2006). Biochemical and functional characterization of an L-amino acid
oxidase isolated from Bothrops pirajai snake venom. Bioorg Med Chem.

[B36] Li Lee M, Chung I, Yee Fung S, Kanthimathi MS, Hong Tan N (2014). Antiproliferative activity of king cobra (Ophiophagus hannah)
venom L-amino acid oxidase. Basic Clin Pharmacol Toxicol.

[B37] Wang H, Joseph JA (1999). Quantifying cellular oxidative stress by dichlorofluorescein
assay using microplate reader. Free Radic Biol Med.

[B38] van Engeland M, Nieland LJ, Ramaekers FC, Schutte B, Reutelingsperger CP (1998). Annexin V-affinity assay: a review on an apoptosis detection
system based on phosphatidylserine exposure. Cytometry.

[B39] Riccardi C, Nicoletti I (2006). Analysis of apoptosis by propidium iodide staining and flow
cytometry. Nat Protoc.

[B40] Costa TR, Burin SM, Menaldo DL, de Castro FA, Sampaio SV (2014). Snake venom L-amino acid oxidases: an overview on their antitumor
effects. J Venom Anim Toxins incl Trop Dis.

[B41] Rodrigues RS, Izidoro LFM, de Oliveira RJ, Sampaio SV, Soares AM, Rodrigues VM (2009). Snake venom phospholipases A2: a new class of antitumor
agents. Protein Pept Lett.

[B42] Bregge-Silva C, Nonato MC, de Albuquerque S, Ho PL, Junqueira de Azevedo ILM, Vasconcelos Diniz MR (2012). Isolation and biochemical, functional and structural
characterization of a novel L-amino acid oxidase from Lachesis muta snake
venom. Toxicon.

[B43] Machado ART, Aissa AF, Ribeiro DL, Costa TR, Ferreira RS, Sampaio SV (2019). Cytotoxic, genotoxic, and oxidative stress-inducing effect of an
l-amino acid oxidase isolated from Bothrops jararacussu venom in a
co-culture model of HepG2 and HUVEC cells. Int J Biol Macromol.

[B44] Ribeiro PH, Zuliani JP, Fernandes CFC, Calderon LA, Stábeli RG, Nomizo A (2016). Mechanism of the cytotoxic effect of l-amino acid oxidase
isolated from Bothrops alternatus snake venom. Int J Biol Macromol.

[B45] Costa TR, Menaldo DL, Prinholato da Silva C, Sorrechia R, de Albuquerque S, Pietro RCLR (2015). Evaluating the microbicidal, antiparasitic and antitumor effects
of CR-LAAO from Calloselasma rhodostoma venom. Int J Biol Macromol.

[B46] Elmore S (2007). Apoptosis: A review of programmed cell death. Toxicol Pathol.

[B47] Zivny J, Klener P, Pytlik R, Andera L (2010). The role of apoptosis in cancer development and treatment:
focusing on the development and treatment of hematologic
malignancies. Curr Pharm Des.

[B48] Poon IKH, Hulett MD, Parish CR (2010). Molecular mechanisms of late apoptotic/necrotic cell
clearance. Cell Death Differ.

[B49] Bueno-da-Silva AEB, Brumatti G, Russo FO, Green DR, Amarante-Mendes GP (2003). Bcr-Abl-mediated resistance to apoptosis is independent of
constant tyrosine-kinase activity. Cell Death Differ.

[B50] Brumatti G, Weinlich R, Chehab CF, Yon M, Amarante-Mendes GP (2003). Comparison of the anti-apoptotic effects of Bcr-Abl, Bcl-2 and
Bcl-x(L) following diverse apoptogenic stimuli. FEBS Lett.

[B51] Yanovich S, Hall RE, Weinert C (1986). Resistance to natural killer cell-mediated cytolysis by a
pleiotropic drug-resistant human erythroleukemia (K562-R) cell
line. Cancer Res.

[B52] Donato NJ, Wu JY, Stapley J, Gallick G, Lin H, Arlinghaus R (2003). BCR-ABL independence and LYN kinase overexpression in chronic
myelogenous leukemia cells selected for resistance to STI571. Blood.

[B53] Moustafa IM, Foster S, Lyubimov AY, Vrielink A (2006). Crystal structure of LAAO from Calloselasma rhodostoma with an
L-phenylalanine substrate: insights into structure and
mechanism. J Mol Biol.

[B54] Samel M, Vija H, Rönnholm G, Siigur J, Kalkkinen N, Siigur E (2006). Isolation and characterization of an apoptotic and platelet
aggregation inhibiting L-amino acid oxidase from Vipera berus berus (common
viper) venom. Biochim Biophys Acta.

[B55] Ande SR, Kommoju PR, Draxl S, Murkovic M, Macheroux P, Ghisla S (2006). Mechanisms of cell death induction by L-amino acid oxidase, a
major component of ophidian venom. Apoptosis.

[B56] Alves RM, Antonucci GA, Paiva HH, Cintra ACO, Franco JJ, Mendonça-Franqueiro EP (2008). Evidence of caspase-mediated apoptosis induced by l-amino acid
oxidase isolated from Bothrops atrox snake venom. Comp Biochem Physiol A Mol Integr Physiol.

[B57] Zhang L, Wei LJ (2007). ACTX-8, a cytotoxic L-amino acid oxidase isolated from
Agkistrodon acutus snake venom, induces apoptosis in Hela cervical cancer
cells. Life Sci.

[B58] Zhang L, Cui L (2007). A cytotoxin isolated from Agkistrodon acutus snake venom induces
apoptosis via Fas pathway in A549 cells. Toxicol In Vitro.

[B59] Jaenisch R, Bird A (2003). Epigenetic regulation of gene expression: how the genome
integrates intrinsic and environmental signals. Nat Genet.

[B60] Baylin SB (2005). DNA methylation and gene silencing in cancer. Nat Clin Pract Oncol.

[B61] Zinkel SS, Ong CC, Ferguson DO, Iwasaki H, Akashi K, Bronson RT (2003). Proapoptotic BID is required for myeloid homeostasis and tumor
suppression. Genes Dev.

[B62] Golks A, Brenner D, Fritsch C, Krammer PH, Lavrik IN (2005). c-FLIPR, a new regulator of death receptor-induced
apoptosis. J Biol Chem.

[B63] Pereira WO, Amarante-Mendes GP (2011). Apoptosis: a programme of cell death or cell
disposal?. Scand J Immunol.

[B64] Fawzy MS, Toraih EA, Ibrahiem A, Abdeldayem H, Mohamed AO, Abdel-Daim MM (2017). Evaluation of miRNA-196a2 and apoptosis-related target genes:
ANXA1, DFFA and PDCD4 expression in gastrointestinal cancer patients: A
pilot study. PLoS One.

[B65] Calin GA, Croce CM (2006). MicroRNA signatures in human cancers. Nat Rev Cancer.

[B66] Hummel R, Hussey DJ, Haier J (2010). MicroRNAs: predictors and modifiers of chemo- and radiotherapy in
different tumour types. Eur J Cancer.

[B67] Rakshit S, Mandal L, Pal BC, Bagchi J, Biswas N, Chaudhuri J (2010). Involvement of ROS in chlorogenic acid-induced apoptosis of
Bcr-Abl+ CML cells. Biochem Pharmacol.

