# Supplementary material for: Bothrops moojeni L-amino acid oxidase induces apoptosis and epigenetic modulation on Bcr-Abl+ cells
Source: J Venom Anim Toxins Incl Trop Dis. 2020 Dec 14;26:e20200123. doi: 10.1590/1678-9199-JVATITD-2020-0123 (PMC7737401; doi:10.1590/1678-9199-JVATITD-2020-0123)
Supplement: Additional file 1. [file 1678-9199-jvatitd-26-e20200123-s1.pdf]

J Venom Anim Toxins incl Trop Dis
https://doi.org/10.1590/1678-9199-JVATITD-2020-0123

Supplementary Material to "*Bothrops moojeni* L-amino acid oxidase induces apoptosis and epigenetic modulation on Bcr-Abl+ cells"

Additional file 1. Parameters of L-amino acid oxidase from *B. moojeni* snake venom purification procedure.

Purification procedure	Protein (mg)	Recovery protein (%)	Total activity (U)*	Specific activity (U/mg)	Purification fold
***B. moojeni* venom**	200	100	45,620	0,228	1
**CM-sepharose**	50	25	25,091	0,502	2,2
**Phenyl-sepharose**	16	8	25,547	1,597	7
**Benzamidine -sepharose**	4	2	11,224	2,806	12,3

*One unit (U) of enzymatic activity is defined as the amount of enzyme that produces 1 µmol H_2O_2/min.
