# Supplementary material for: Bothrops moojeni L-amino acid oxidase induces apoptosis and epigenetic modulation on Bcr-Abl+ cells
Source: J Venom Anim Toxins Incl Trop Dis. 2020 Dec 14;26:e20200123. doi: 10.1590/1678-9199-JVATITD-2020-0123 (PMC7737401; doi:10.1590/1678-9199-JVATITD-2020-0123)
Supplement: Additional file 2. [file 1678-9199-jvatitd-26-e20200123-s2.pdf]

J Venom Anim Toxins incl Trop Dis
https://doi.org/10.1590/1678-9199-JVATITD-2020-0123

Supplementary Material to "*Bothrops moojeni* L-amino acid oxidase induces apoptosis and epigenetic modulation on Bcr-Abl⁺ cells"

Additional file 2. Oligonucleotide sequences used for quantification of target gene expression.

Target gene	Oligonucleotide sequence (5' to 3')
BID	F: GCT TCC AGT GTA GAC GGA GC
	R: GTG CAG ATT CAT GTG TGG ATG
FADD	F: GTT CTC CTT CTC TGT GTT CT
	R: CTC AAG TTC CTA TGC CTC
β-ACTIN	F: GCC CTG AGG CAC TCT TCC A
	R: CCA GGG CAG TGA TCT CCT TCT
B2M	F: CCA GCG TAC TCCC AAA GAT TCA
	R: TGG ATG AAA CCC AGA CAC ATA G
