# Supplementary material for: Bothrops moojeni L-amino acid oxidase induces apoptosis and epigenetic modulation on Bcr-Abl+ cells
Source: J Venom Anim Toxins Incl Trop Dis. 2020 Dec 14;26:e20200123. doi: 10.1590/1678-9199-JVATITD-2020-0123 (PMC7737401; doi:10.1590/1678-9199-JVATITD-2020-0123)
Supplement: Additional file 3. [file 1678-9199-jvatitd-26-e20200123-s3.pdf]

J Venom Anim Toxins incl Trop Dis
https://doi.org/10.1590/1678-9199-JVATITD-2020-0123

Supplementary Material to "*Bothrops moojeni* L-amino acid oxidase induces apoptosis and epigenetic modulation on Bcr-Abl$^+$ cells"

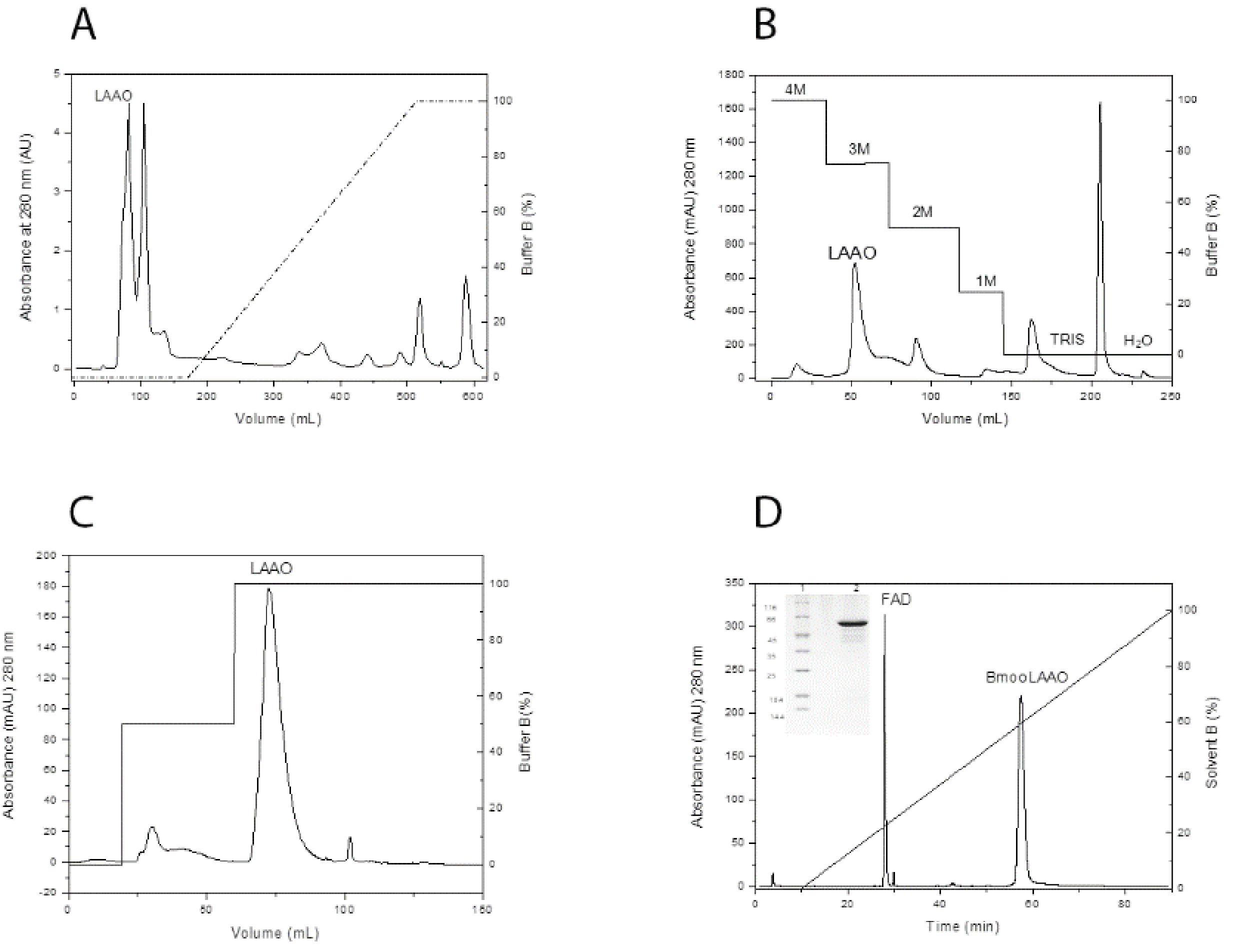

Additional file 3. Purification of the L-amino acid oxidase BmooLAAO-I from *Bothrops moojeni* snake venom. **(A)** Chromatographic profile of 200 mg of desiccated venom applied onto a CM-sepharose column previously equilibrated with 0.05 M NH_4NaHCO_3 buffer, pH 8.0, and eluted with a concentration gradient of up to 1.0 M of the same buffer (buffer B) at room temperature. The LAAO-active fraction eluted in the first peak. **(B)** Chromatographic profile of the pooled and concentrated LAAO-active fraction obtained in (A), applied onto a phenyl-sepharose column previously equilibrated with 0.02 M Tris–HCl buffer, pH 7.6 (buffer B) containing 4 M NaCl, and eluted with a discontinuous gradient of 4–0 M NaCl in the same buffer. **(C)** Chromatographic profile of the pooled and concentrated LAAO-active fraction obtained in (B), applied onto a benzamidine sepharose column pre-equilibrated with 20 mM Tris-HCl, pH 7.4 (buffer A), and eluted with a step gradient of 20 mM Tris-HCl containing 1.0 M NaCl, pH 7.4 (buffer B). **(D)** Purity of the active enzyme BmooLAAO-I assessed by SDS-PAGE (inset) and reversed-phase HPLC.
