# Supplementary material for: Bothrops moojeni L-amino acid oxidase induces apoptosis and epigenetic modulation on Bcr-Abl+ cells
Source: J Venom Anim Toxins Incl Trop Dis. 2020 Dec 14;26:e20200123. doi: 10.1590/1678-9199-JVATITD-2020-0123 (PMC7737401; doi:10.1590/1678-9199-JVATITD-2020-0123)
Supplement: Additional file 4. [file 1678-9199-jvatitd-26-e20200123-s4.pdf]

J Venom Anim Toxins incl Trop Dis
https://doi.org/10.1590/1678-9199-JVATITD-2020-0123

Supplementary Material to "*Bothrops moojeni* L-amino acid oxidase induces apoptosis and epigenetic modulation on Bcr-Abl[+] cells"

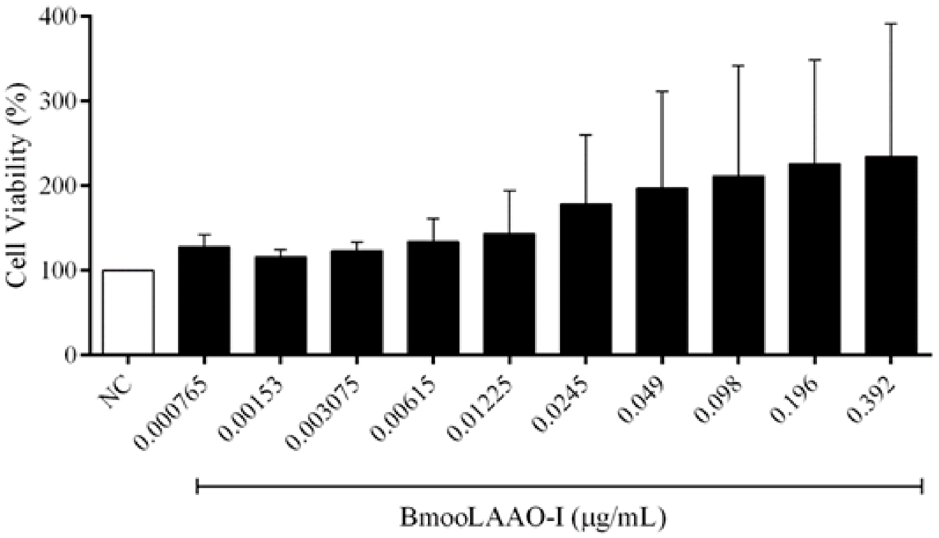

Additional fille 4. Cytotoxicity of BmooLAAO-I towards PBMC, at 24 h post-treatment. Results are expressed as mean ± standard deviation of the percentage of cell viability from three samples assayed in triplicate. NC: negative control (untreated cells). $p > 0.05$ *vs*. NC (one-way ANOVA combined with the Tukey's *post-hoc* test).
