# Supplementary material for: Bothrops moojeni L-amino acid oxidase induces apoptosis and epigenetic modulation on Bcr-Abl+ cells
Source: J Venom Anim Toxins Incl Trop Dis. 2020 Dec 14;26:e20200123. doi: 10.1590/1678-9199-JVATITD-2020-0123 (PMC7737401; doi:10.1590/1678-9199-JVATITD-2020-0123)
Supplement: Additional file 5. [file 1678-9199-jvatitd-26-e20200123-s5.pdf]

J Venom Anim Toxins incl Trop Dis
https://doi.org/10.1590/1678-9199-JVATITD-2020-0123

Supplementary Material to "*Bothrops moojeni* L-amino acid oxidase induces apoptosis and epigenetic modulation on Bcr-Abl$^+$ cells"

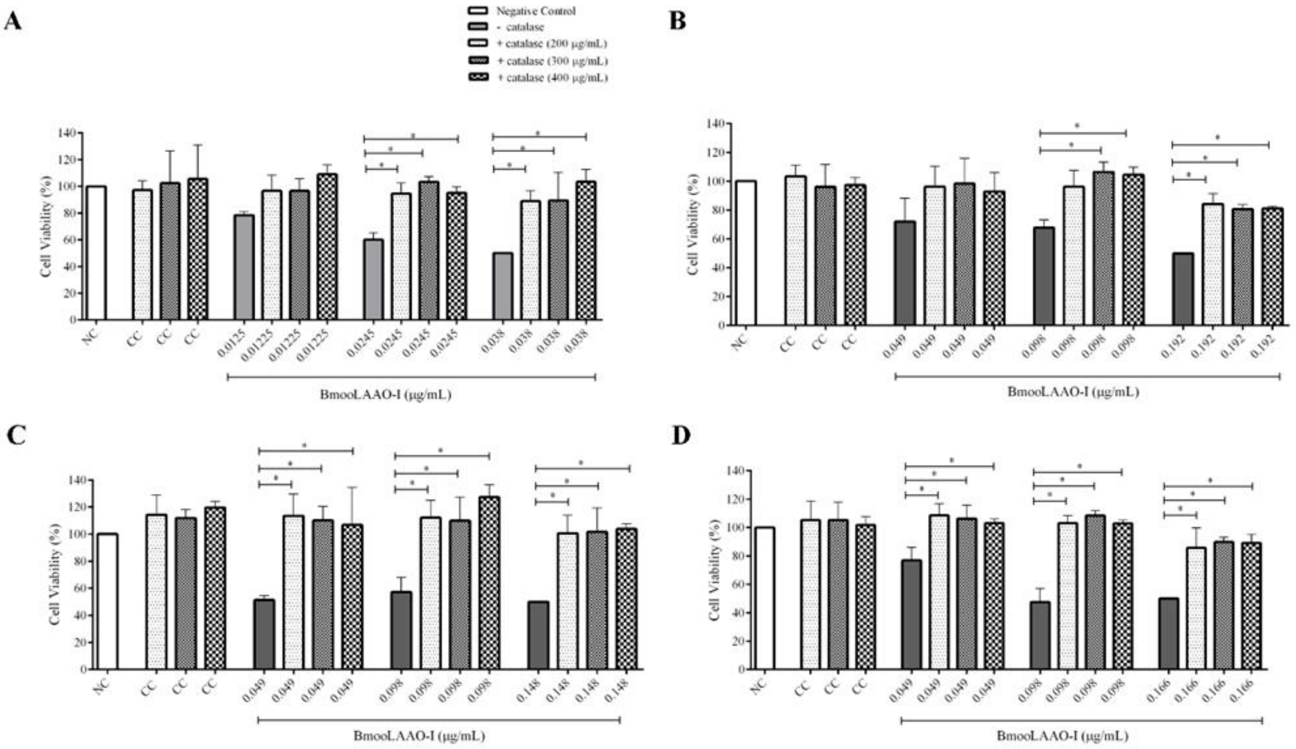

Additional file 5. Cytotoxicity of BmooLAAO-I towards tumor cell lines in the presence of 200-400 µg/mL of catalase. **(A)** HL-60 cells, **(B)** HL-60.Bcr-Abl cells, **(C)** K562-S cells, and **(D)** K562-R cells. Results are expressed as mean ± standard deviation of the percentage of cell viability from three independent experiments assayed in triplicate. Cells were treated with the toxin for 24 h. NC: negative control (untreated cells); CC: catalase control (cells treated with catalase only). *$p < 0.05$ *vs.* (−) catalase (one-way ANOVA combined with the Tukey's *post-hoc* test).
