# Supplementary material for: Bothrops moojeni L-amino acid oxidase induces apoptosis and epigenetic modulation on Bcr-Abl+ cells
Source: J Venom Anim Toxins Incl Trop Dis. 2020 Dec 14;26:e20200123. doi: 10.1590/1678-9199-JVATITD-2020-0123 (PMC7737401; doi:10.1590/1678-9199-JVATITD-2020-0123)
Supplement: Additional file 6. [file 1678-9199-jvatitd-26-e20200123-s6.pdf]

J Venom Anim Toxins incl Trop Dis
https://doi.org/10.1590/1678-9199-JVATITD-2020-0123

Supplementary Material to "*Bothrops moojeni* L-amino acid oxidase induces apoptosis and epigenetic modulation on Bcr-Abl^{+} cells"

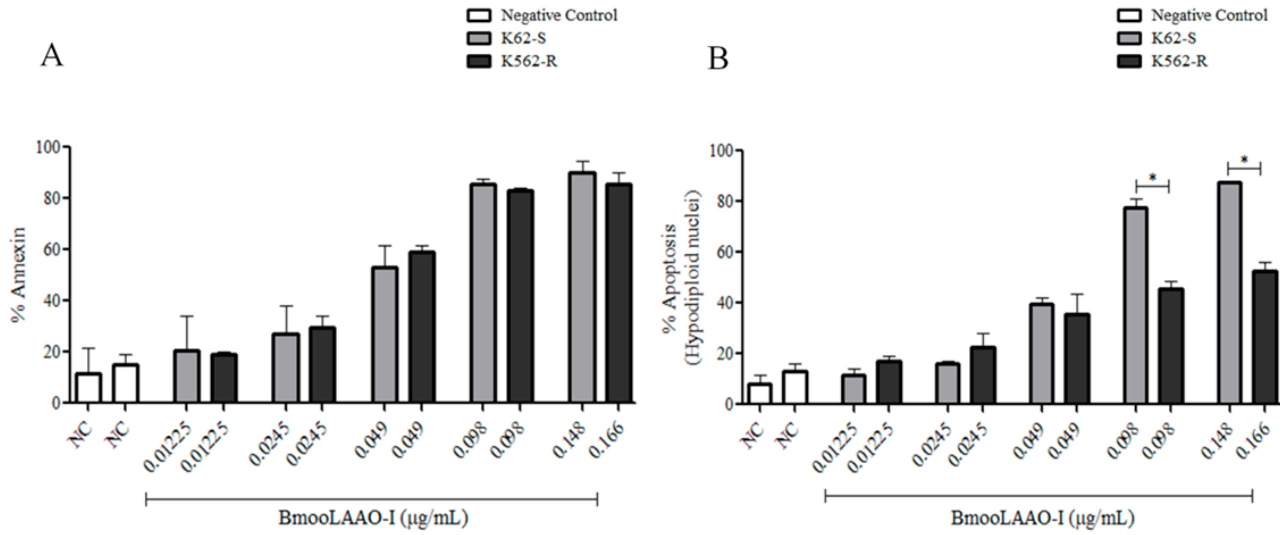

Additional file 6. Comparison between BmooLAAO-I-induced apoptosis levels in K562-S and K562-R cells. **(A)** Total percentage of annexin-V-stained cells. **(B)** Percentage of hipodiploid nuclei. Results are expressed as mean ± standard deviation of three independent experiments. NC: negative control (untreated cells). *p < 0.05 *vs.* NC (one-way ANOVA combined with the Tukey's *post-hoc* test).
