# Supplementary material for: Bothrops moojeni L-amino acid oxidase induces apoptosis and epigenetic modulation on Bcr-Abl+ cells
Source: J Venom Anim Toxins Incl Trop Dis. 2020 Dec 14;26:e20200123. doi: 10.1590/1678-9199-JVATITD-2020-0123 (PMC7737401; doi:10.1590/1678-9199-JVATITD-2020-0123)
Supplement: Additional file 7. [file 1678-9199-jvatitd-26-e20200123-s7.pdf]

J Venom Anim Toxins incl Trop Dis
https://doi.org/10.1590/1678-9199-JVATITD-2020-0123

Supplementary Material to "*Bothrops moojeni* L-amino acid oxidase induces apoptosis and epigenetic modulation on Bcr-Abl^{+} cells"

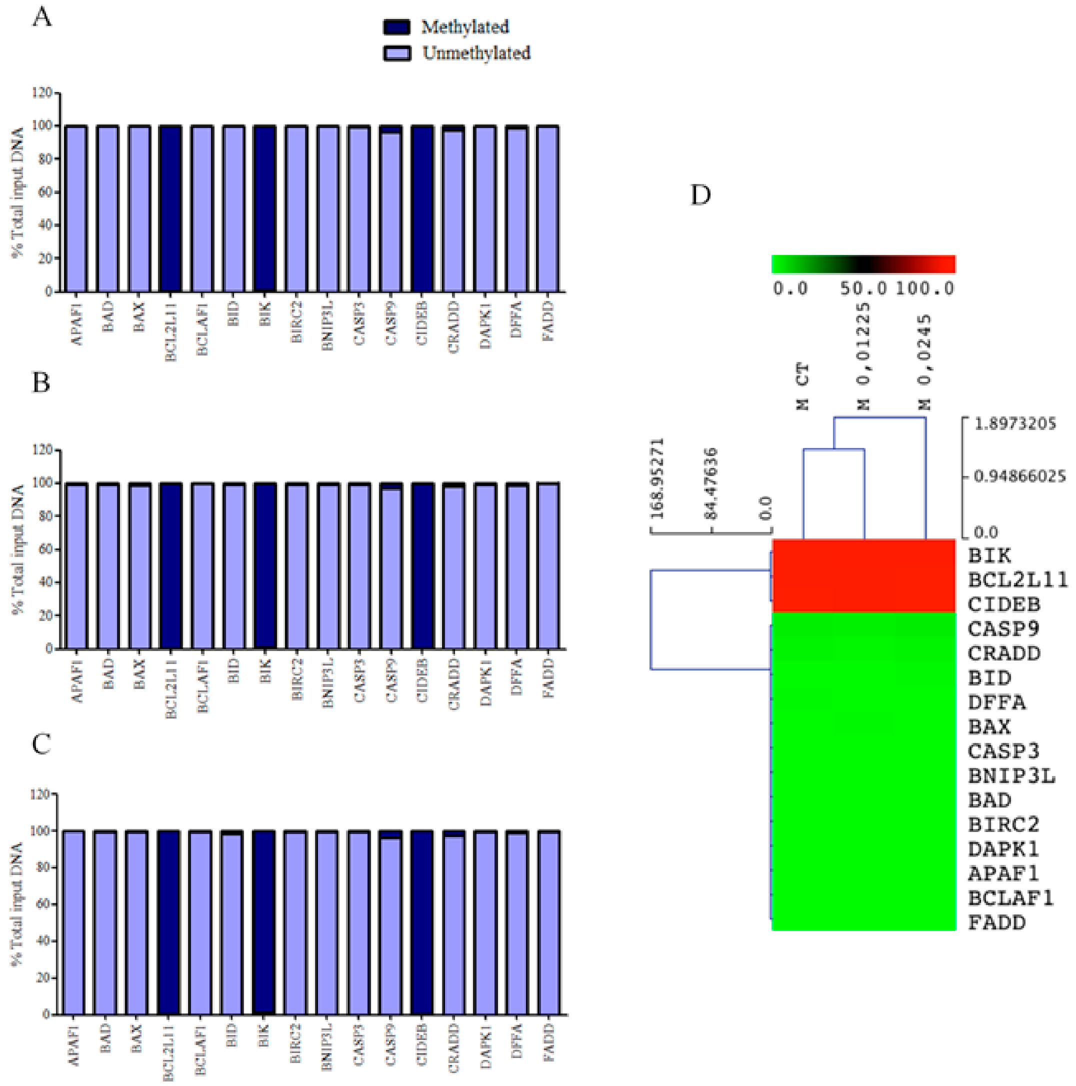

Additional file 7. BmooLAAO-I did not alter the methylation pattern of apoptosis-related genes in K562-R cells. The percentage of methylation of the promoter region of apoptosis-related genes was quantified by real-time PCR in cells treated with BmooLAAO-I for 24 h. **(A)** Untreated cells (negative control). **(B)** Cells treated with the toxin at 0.01225 µg/mL. **(C)** Cells treated with the toxin at 0.0245 µg/mL. **(D)** Heatmap of sample clustering according to the percentage of methylated DNA. The horizontal bar in the top of the heatmap represents the color scale of percentage of methylation ranging from 0-100%.
