# Supplementary material for: Bothrops moojeni L-amino acid oxidase induces apoptosis and epigenetic modulation on Bcr-Abl+ cells
Source: J Venom Anim Toxins Incl Trop Dis. 2020 Dec 14;26:e20200123. doi: 10.1590/1678-9199-JVATITD-2020-0123 (PMC7737401; doi:10.1590/1678-9199-JVATITD-2020-0123)
Supplement: Additional file 8. [file 1678-9199-jvatitd-26-e20200123-s8.pdf]

J Venom Anim Toxins incl Trop Dis
https://doi.org/10.1590/1678-9199-JVATITD-2020-0123

Supplementary Material to "*Bothrops moojeni* L-amino acid oxidase induces apoptosis and epigenetic modulation on Bcr-Abl+ cells"

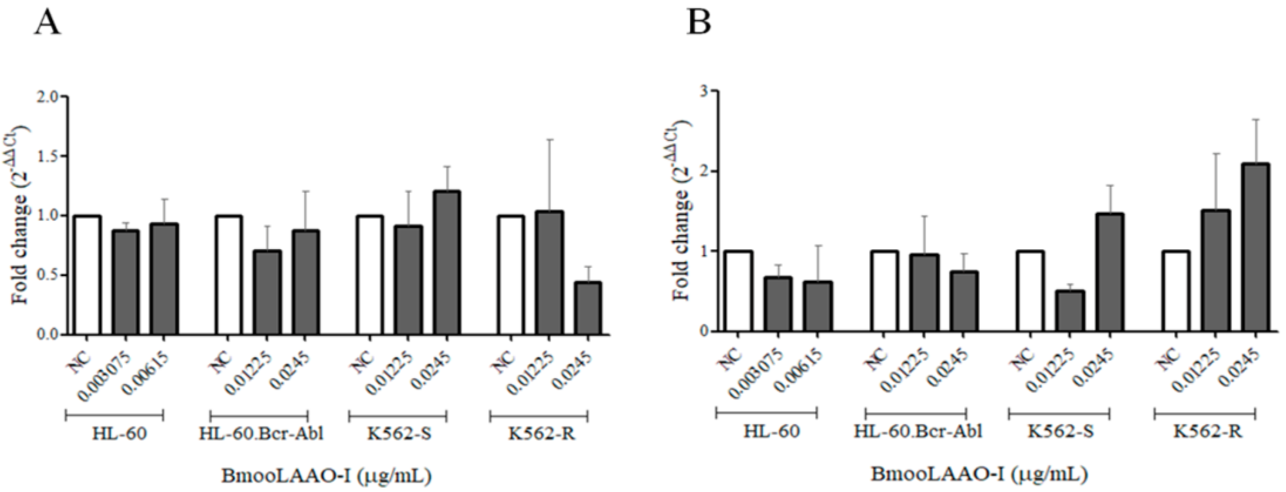

Additional file 8. ApoptomiRs expression in tumor cell lines treated with BmooLAAO-I. Real-time PCR quantification of the apoptomiRs **(A)** miR-15a and **(B)** has-let-7d in HL-60, HL-60.Bcr-Abl, K562-S, and K562-R cells treated for 24 h with BmooLAAO-I at sublethal concentrations. Results are expressed as mean fold change ± standard deviation of three independent experiments. NC: negative control (untreated cells). *$p < 0.05$ *vs.* NC (one-way ANOVA followed by the Tukey's *post-hoc* test).
